# Innate and adaptive immune patterns in hospitalised COVID-19 patients: a framework for precision monitoring in viral and inflammatory syndromes

**DOI:** 10.3389/fimmu.2025.1683748

**Published:** 2026-02-04

**Authors:** Salvatore Corrao, Raffaella Mallaci Bocchio, Salvatore Scibetta, Antonella Montalbano, Giuseppe Natoli, Luigi Calvo, Francesco Gervasi, Christiano Argano, Internal Medicine IGR COVID-19 Investigators

**Affiliations:** 1 Department of Internal Medicine, National Relevance and High Specialization Hospital Trust Azienda di Rilievo Nazionale ad Alta Specializzazione (A.R.N.A.S.) Civico, Di Cristina, Benfratelli, Palermo, Italy; 2 Department of Health Promotion Sciences, Maternal and Infant Care, Internal Medicine and Medical Specialties. [PROMISE], University of Palermo, Palermo, Italy; 3 Institute for Biomedical Research and Innovation (IRIB), National Research Council (CNR), Palermo, Italy; 4 Specialized Laboratory of Oncology, National Relevance and High Specialization Hospital Trust Azienda di Rilievo Nazionale ad Alta Specializzazione (A.R.N.A.S.) Civico, Di Cristina, Benfratelli, Palermo, Italy

**Keywords:** COVID-19 patients, immune system, innate immunity, intensive care unit, natural killer (NK) cells, T helper (CD4)

## Abstract

**Background:**

Understanding immune responses in viral infections such as COVID-19 is crucial for identifying patients at risk of clinical deterioration. Profiling innate and adaptive immune components may provide a basis for precision monitoring and personalised management strategies in infectious and inflammatory syndromes.

**Methods:**

In a prospective observational study, 150 patients were enrolled from 30 March to 15 April 2022, hospitalised for COVID-19 at a tertiary Internal Medicine COVID Unit. Flow cytometry analysis of peripheral blood quantified 34 immune subpopulations. Patients were stratified into immunophenotypic groups based on deficiencies in T helper (CD4), cytotoxic T (CD8), natural killer (NK), and plasma cells. Clinical outcomes were analysed in relation to these immune profiles using multivariate logistic regression.

**Results:**

Of the 150 patients (mean age 64.7 years, 59.1% male), 7.8% required ICU admission and 9.1% died. Lymphopenia (<1200 cells/µL) was observed in 84%, with 61.1% showing CD4+ T cell deficiency, 32.2% NK cell deficiency, 46% CD8+ T cell reduction, and 26.6% decreased plasma cells. Notably, 10% had concurrent CD4 and NK cell deficiencies. While low plasma cell counts were not associated with worse outcomes, NK cell deficiency was linked to a threefold increased risk of death or ICU transfer (OR 3.19, p<0.03), and CD4+ T cell reduction was associated with a 5.23-fold increase (p<0.037). The combination of low CD4 and NK cells resulted in a 9.5-fold higher risk of adverse outcome, independently of age and sex.

**Conclusions:**

Specific immune profiles, particularly reductions in CD4+ and NK cells, are strong predictors of mortality or ICU admission in hospitalized COVID-19 patients. These findings suggest that rapid and clinically feasible tool may support precision monitoring and personalised management in viral and inflammatory syndromes beyond COVID-19. This study advocates for the integration of immune profiling into the framework of precision medicine.

## Introduction

The COVID-19 pandemic, caused by the emergence of the novel severe acute respiratory syndrome coronavirus 2 (SARS-CoV-2), has significantly transformed the landscape of global health. Beyond its immediate clinical effects, it has exposed weaknesses in public health systems and highlighted the importance of understanding host-pathogen interactions at the immunological level. Although the pandemic is now reaching a phase of epidemiological stabilisation, its scientific and clinical lessons continue to be relevant, particularly regarding the immune system’s crucial role in shaping disease outcomes. A key feature of COVID-19 is its significant variability in clinical presentation ranging from asymptomatic infection to life-threatening respiratory failure. This diversity has consistently been associated with differences in the host immune response, particularly the coordination (or dysregulation) between innate and adaptive immunity (1, 2). It is now well recognised that SARS-CoV-2 causes a complex interaction between viral replication and immune activation. In mild cases, a balanced and prompt response involving natural killer (NK) cells, T lymphocytes, and antibody-producing plasma cells aids in viral clearance and recovery (3–5). Conversely, in severe cases, immune dysregulation can result in hyperinflammation, cytokine storm, lymphocyte depletion, and ultimately multi-organ failure (6, 7). Of particular interest is the observation that reductions in specific immune cell subsets namely CD4+ helper T cells, CD8+ cytotoxic T cells, and NK cells—are frequently linked to severe disease and higher mortality (8–12).

These immune changes not only impair viral clearance but also diminish the effectiveness of immunomodulatory therapies, highlighting their importance in clinical management. Furthermore, the exhaustion of T cell function, impaired cytotoxic responses, and altered plasma cell dynamics have been documented in critically ill patients, indicating deeper immune dysfunction beyond just numerical reduction (13–15). The pandemic has also sparked a renewed interest in the clinical use of immunophenotyping (16). Multiparametric flow cytometry (MPFC) enables the rapid and comprehensive characterisation of immune cell populations using minimal sample volumes, providing a real-time insight into the host’s immunological state (17). By utilising this approach, it becomes possible to identify specific immune profiles linked to different clinical outcomes, laying a groundwork for early risk assessment and personalised interventions. In previous studies, our group identified distinct immunological clusters among non-ICU hospitalised COVID-19 patients, with the most compromised cluster characterised by concurrent reductions in CD4+, CD8+, NK cells, and plasma cells exhibiting the highest mortality (18–20). These findings suggested that the immunological phenotype, rather than viral burden alone, may be a key determinant of prognosis. The present study expands on this work by examining, in a cohort of 150 hospitalised patients, how various patterns of innate and adaptive immune cell depletion relate to clinical outcomes. Importantly, although the acute crisis phase of COVID-19 may be subsiding, the methodological and conceptual implications of this research go well beyond the disease itself. The ability to identify vulnerable immune profiles offers potential not only in managing future respiratory viral infections but also in other inflammatory, post-infectious, or immunosuppressed conditions where early prognostication is essential.

Thus, this study aims to provide both a deeper understanding of immune dysregulation in COVID-19 and a broader framework for precision immunomonitoring in patients affected by both current and future viral or inflammatory syndromes. By integrating immunological data into clinical decision-making, we move one step closer to a truly personalised model of care one that does not merely react to disease, but anticipates and pre-empts its progression.

## Methods

### Data collection

From March 30 to April 15, 2022, 150 patients with confirmed COVID-19 (positive RT-PCR for SARS-CoV-2 and presence of typical radiological signs) admitted to the COVID Unit of the Department of Clinical Medicine at the National and Highly Specialized Hospital Arnas Civico-Di Cristina-Benfratelli of Palermo were consecutively enrolled. Written informed consent was routinely obtained from all patients at admission. Data were collected regarding the patient’s medical history, clinical, biological and immunology, and the days elapsed between the nasopharyngeal test positivity and hospitalization.Patients were eligible if they had: confirmed SARS-CoV-2 infection by RT-PCR, radiological evidence consistent with COVID-19 pneumonia.

Exclusion criteria included: known hematologic malignancy, active immunosuppressive therapy, chronic immunodeficiency, or refusal to provide consent. Blood samples were collected upon hospital admission, with a median of 7 days (IQR 5–10) from symptom onset.

### Flow cytometry

The gating strategy for lymphocyte subset identification, following the guidelines of the Human ImmunoPhenotyping Consortium was adopted (18).

A six- to eight-colour flow cytometry assay was developed covering 34 different immune cell subgroups from only 2 ml of human peripheral blood treated with anticoagulants (EDTA). The MPFC immunophenotyping assay was used to stain whole blood samples directly. This technique detects all circulating immune cells and reduces the preparation steps required for flow cytometry. The direct staining procedure minimizes effort and variation in sample preparation and is a time saver, an additional prerequisite for easy clinical application, requiring less than 20 minutes of hands-on time (18).

All lymphocyte populations, including subpopulations T (T), T-helper (TH), T-cytotoxic (TC), Natural Killer (NK), T/NK, and B, were determined in both percentage and absolute counts.

All monoclonal antibodies were purchased from Beckman-Coulter (Miami, Florida). Whole blood samples were incubated with monoclonal antibodies for 15 min at room temperature and lysed with ammonium chloride for 20 min at 4 °C by lyse- no-wash method and at least 25000 total events, excluding aggregates of cells and debris, were acquired on Beckman - Coulter (Miami Fla) Naviostm flow cytometer. The analysis of the acquired samples was performed by Kaluza analysis 2.1 Beckman -Coulter software (Miami, Fla), with SS/CD45 gate for determination of lymphocyte populations and subpopulations and by double gate CD19/SS and CD38/SS for determination of total and secreting plasma cells (21).

### Microbiology

Molecular diagnostics of SARS-Cov-2 was performed at the Virology Laboratory of the Department of Microbiology, Arnas Civico di Palermo, using the detection of single-stranded positive viral RNA from nasopharyngeal swabs by (RT-PCR) reverse polymerase chain reaction (Elitech Ingenius - Arrow SeGeneNimbus) (22).

### Statistical analysis

Data are presented as percentages in the case of categorical variables, as mean (95% confidence interval) and as median (interquartile range Q1 -Q3) in the case of quantitative variables. Two-way analysis of variance (ANOVA) was used to compare all variables in multiple ways. The significance level used was the two-tailed 0.05. Univariate and multivariate logistic regressions were performed to assess the relationship between the dichotomous outcome and the variables examined. The odds ratios (ORs) were calculated with the respective intervals of confidence intervals at 95% (CI at 95%) values of p -value. For all analyses, STATA version 17 (StataCorp.2021, College Station, TX, USA: StataCorp LP) was used for database management and analysis.

## Results

A total of 150 patients with a confirmed diagnosis of COVID-19 were eligible for this analysis; among them, 59.1% were male, with an average age of 64.7. **Table 1** shows the clinical and laboratory characteristics of the analysed population. The average hospital stay for each patient was 20 days. 7.8% of patients were transferred to the intensive care unit, while 9.1% died. The number of lymphocytes was 879,71 cells/uL (505,41 - 1254,01) at admission, while the number almost doubled to 1529,32 cells/uL (480,30-2578,34) upon discharge. MPFC immunophenotyping analysis (**Table 2**) showed that 84% of patients had a total lymphocyte count of less than 1200 cells/µL with a mean lymphocyte count of 704.76 cells/µL (622.61 to 786.92). 61.1% of patients had a T helper lymphocyte count <500 cells/µL, while a natural killer lymphocyte deficiency was found in 32.2% of patients. Several plasma cells <1 cells/µL were found in 26.6% of the sample, and cytotoxic T lymphocytes <200 cells/µL in 46% of the patients. Ten per cent had a deficit of T helper and natural killer lymphocytes. Based on the data obtained, the sample was divided into four groups relative to immunological characteristics:

**Table 1:** Clinical and laboratory variables of the population analysed.

Men _(%)_	59,1
Age§	64,7 (55 - 76)
Inpatient _Days§_	20 (13 - 30)
Transfer to ICU/UTIR _(%)_	7,8
Death _(%)_	9,1
^Hb*^ (gr/dl) at admission	12,94 (12,59 - 13,30)
^Hb*^ (gr/dl) at discharge	12,14 (11,80 - 12,47)
^GB*^ (cell/uL) at admission	7817,86 (6978,35 - 8657,37)
^GB*^ (cells/uL) at discharge	9364,89 (8090,74 - 10639,05)
^NEUT*^ (cell/uL) at admission	4212,23 (3391,72 - 5032,75)
^NEUT*^ (cells/uL) at discharge	4283,70 (3420,21 - 5147,19)
^LINF*^ (cell/uL) at admission	879,71 (505,41 - 1254,01)
^LINF*^ (cells/uL) at discharge	1529,32 (480,30 - 2578,34)
^PLT*^ (cells/µL) at admission	258,51 (241,10 - 275,91)
^PLT*^ (cells/µL) at discharge	279,77 (256,05 - 303,49)
^PCR*^ (mg/dl) at admission	5,94 (5,00 - 6,87)
^PCR*^ (mg/dl) at discharge	2,79 (1,83 - 3,75)
^Albumin*^ (g/dl) at admission	3,68 (3,56 - 3,79)
^LDH*^ (mg/dl) at admission	300,31 (279,87 - 320,74)
^Fibrinogen*^ (mg/dl) at admission	543,89 (514,33 - 573,44)
^D-Dimer*^ (ng/ml) at admission	2236,62 (1449,96 - 3023,27)
^D-Dimer*^ (ng/ml) at discharge	2174,48 (984,43 - 3364,53)
^Ferritin*^ (ug/L) at admission	687,02 (426,73 - 947,32)
^IL-6*^ (pg/ml) at admission	39,19 (25,24 - 53,14)

*Data are reported as mean (95% confidence interval).

§Data are reported as median (interquartile range Q 1-Q3).

^Hb,^ haemoglobin; ^GB,^ white blood cells; ^NEUT,^ neutrophils; ^LINF,^ lymphocytes; ^PLT,^ platelets; ^PCR,^ c- reactive protein; ^LDH,^ lactic dehydrogenase; ^IL -6,^ interleukin 6.

**Table 2:** Cytofluorimetric variables of the analysed population.

Total T lymphocytes*	704,76 (622,61 - 786,92)
T lymphocytes < 1200 cells/µL (%)	84
T Helper Lymphocytes*	481,48 (429,61 - 533,35)
Helper T lymphocytes < 500 cells/µL (%)	61,1
Natural killer lymphocytes*	173,53 (149,58-197,48)
Natural killer lymphocytes < 100 cells/µL (%)	32,2
Deficiency of innate immunity1 (%)	26,2
Plasma cells*	7,99 (5,54 - 10,44)
Plasma cells < 1 cells/µL (%)	26,6
Cytotoxic T lymphocytes*	273,96 (239,78 - 308,14)
Cytotoxic T lymphocytes < 200 cells/µL (%)	46
Deficiency of additive immunity (%)	10

*Data are reported as mean (95% confidence interval).

^1^Deficiency of innate immunity, understood as the simultaneous deficiency of natural killer (<100 cells/µL) and helper T lymphocytes (<500 cells/µL).

^2^Deficiency of adaptive immunity, understood as the simultaneous deficit of cytotoxic T lymphocytes (< 200 cells/µL) and plasma cells (< 1 cell/µL).

Group 1 ↓NK, patients with Natural Killer (NK) cells below the normal range (100–1200 cells/µL).
Group 2 ↓CD4, patients with T Helper (CD4) Lymphocytes below the normal range (500–2000 cells/µL).
Group 3 ↓TC, patients with cytotoxic T Lymphocytes (TC) below the normal range (200–1200 cells/µL).
Group 4 ↓PLC, patients with plasma cells (PLC) below the normal range (1–11 cells/µL).

**Figure 1:**
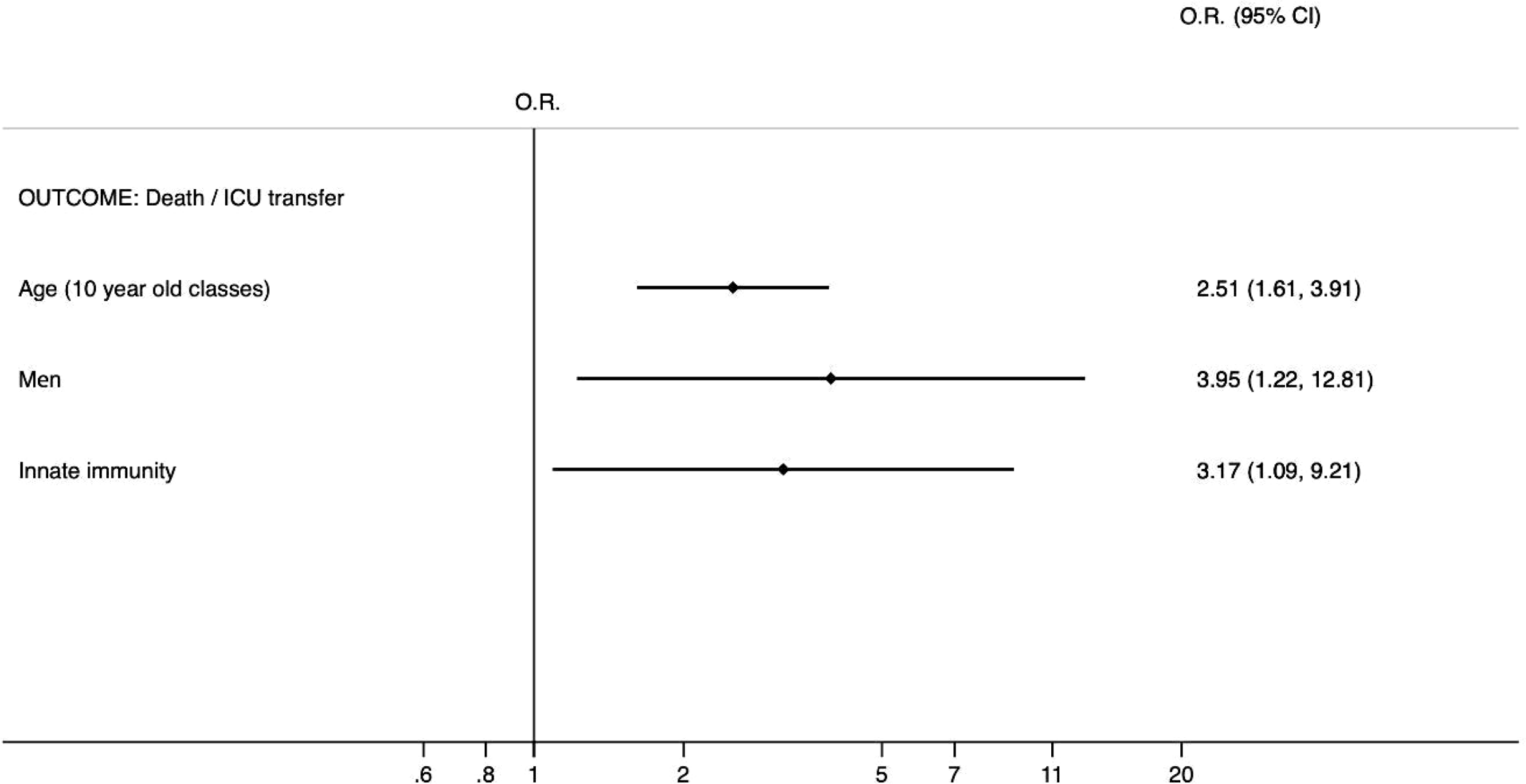
Final multivariate logistic regression between innate immunity (simultaneous reduction of both CD4 and NK) and outcome (death/ICU transfer) independently from age and sex. The following variables related to the chronic diseases were not included into the model because of high p-values. Comorbidities were excluded from the final multivariate model because they did not remain indipendently associated with the outcome after adjustment. Hypertension O.R. 1.64; 95% C.I. (0.39-6.94); p 0.500, Type 2 Diabetes mellitus O.R. 1.35; 95% C.I. (0.46-3.94); p 0.587, Cerebrovascular Disease O.R. 1.17; 95% C.I. (0.37-3.65); p 0.790, Chronic Renal Disease O.R. 1.43; 95% C.I. (0.41-4.98); p 0.576, Obesity O.R. 1.64; 95% C.I. (0.52-5.16); p 0.397.

The analysis of clinical and laboratory characteristics of each group revealed significant differences among the four groups in the following variables: neutrophils at admission and discharge, PCR at admission and discharge, albumin, LDH, and fibrinogen, as shown in **Table 3**. The lower levels of inflammatory markers observed in the ↓PLC group may reflect either a milder disease phenotype or a different phase of the immune response (the convalescent phase), warranting further investigation. The main comorbidities found were arterial hypertension, the most represented in the four groups, along with obesity, type 2 diabetes mellitus, chronic cerebrovascular disease and chronic renal failure, as shown in **Table 4**. In group 2, a higher percentage of subjects with hypertension was observed. Univariate analysis, adjusted for age and sex, showed that reduced cytotoxic T lymphocyte values increased the risk of death or ICU admission by 2.54-fold (odds ratio 2.54). However, this value was not significant (p<0.089). The reduction in the number of plasma cells (odds ratio 0.96) was also not significant (p<0.946) relative to the risk of death or ICU admission. In the group of patients with a reduction in natural killer lymphocytes, the risk of death or admission to non-intensive care unit is increased 3-fold (odds ratio 3.19). This value turns out to be statistically significant (p< 0.03). CD4 reduction also appears to predict death or admission to the intensive care unit in a statistically significant manner (odds ratio 5.23, p<0.037). Reduced innate immunity (simultaneous reduction of both CD4 and NK) increases the likelihood of death or ICU transfer by 9.5-fold, as shown by multivariate logistic regression, adjusted for age and sex. All three variables are found to be predictive of mortality or ICU admission. Innate immunite was included in all models, while comorbidities were excluded from the final multivariate model because they did not remain indipendently associated with the outcome after adjustment (**Figure 1**).

**Table 3:** Comparison of clinical and laboratory variables in the four groups.

Variable	Group 1 ↓NK (n=48)	Group 2 ↓CD4 (n=91)	Group 3 ↓TC (n=69)	Group 4 ↓PL C (n=40)	P
^Hb*^ (gr/dl) at admission	12.85 (12.21 - 13.48)	13.06 (12.57 - 13.54)	13.11 (12.58 - 13.65)	13.27 (12.64 - 13.90)	0.559
^Hb*^ (gr/dl) at discharge	11.64 (10.96 - 12.33)	12.13 (11.67 - 12.59)	12.15 (11.60 - 12.70)	12.29 (11.67 - 12.91)	0.299
^GB*^(cell/uL) at admission	8925.21 (6918 - 10932)	8198.82 (6903 - 9493)	8188.00 (6691- 9684)	7395.92 (5563- 9228)	0.548
^GB*^ (cells/uL) at discharge	9542.39 (7773-11311)	10506.82 (8451-12562)	9919.16 (8422 - 11415	9295.25 (5115 - 13474)	0.287
^NEUT*^ (cell/uL)at admission	5853.92	4968.47	5402.97	4016.45	**0.019**
	(3845-7862)	(3761 - 6175)	(3858- 6947)	(2812 -5220)	
^NEUT*^(cells/uL)atdischarge	6162.20 (4187- 8136)	5406.48 (4095- 6717)	5763.79 (4106- 7421)	3523.13 (2243- 4803)	**0.002**
^LINF*^ (cell/uL)at admission	728.24 (516 - 939)	969.85 (364- 1575)	667.87 (496- 839)	1367.27 (-16.37-2750)	0.281
^LINF*^(cells/uL) at discharge	929.03 (693- 1164)	1797.89 (51- 3544)	841.92 (634- 1049)	3038.72 (-910- 6987)	0.162
^PLT*^ (cells/µL)at admission	273.28	250.58	252.80	224.89	**0.035**
	(238- 307)	(227- 273)	(227- 278)	(196- 253)	
^PLT*^ (cells/µL) at discharge	256.06 (220- 291)	267.30 (239 - 294)	275.71 (241- 309)	270.12 (208- 331)	0.609
^PCR*^ (mg/dl)	7.07	6.93	7.24	4.39	**0.018**
at admission	(5.28 - 8.85)	(5.70 - 8.15)	(5.81 - 8.67)	(2.76 - 6.01)	
^PCR*^ (mg/dl) at discharge	2.68 (1.24 - 4.12)	3.86 (2.35 - 5.37)	3.42 (2.00 - 4.84)	1.89 (0.77 - 3.02)	0.055
Albumin* _(g/dl)_ at admission	3.59 (3.40 - 3.77)	3.58 (3.44 - 3.72)	3.50 (3.36 - 3.63)	3.79 (3.56 - 4.02)	**0.036**
^LDH*^ (mg/dl) at admission	343.57 (301.33 -385.81)	318.30 (290.49-346.11)	346.10 (310.95 -381.25)	276.34 (238.49 - 314.18)	**<0.001**
^Fibrinogen*^ (mg/dl) at admission	580.58 (530.24 - 630.91)	574.93 (540.26 - 609.61)	594.19 (553.00 - 635.38)	504.13 (451.93 -556.33)	**0.016**
D-Dimer* (ng/ml) at admission	3111.84 (1258.60 - 4965.09)	2366.72 (1370.2-3363.16)	2453.67 (1182.71-3724.6)	1158.76 (733.08 -1584.4)	0.304
D-Dimer*(ng/ml) upon discharge	1172.93 (693.29 -1652.57)	3056.96 (1120.32-4993.6)	2218.44 (847.02 -3589.86)	982.22 (309.82 -1654.62)	0.096
Ferritin* _(ug/L)_ at admission	1154.37 (381.45 -1927.29)	816.04 (421.45 -1210.62)	858.92 (359.58 -1358.26)	567.93 (286.45 -849.41)	0.149
^IL-6*^ (pg/ml) at admission	54.11 (20.91 -87.30)	39.57 (25.31 - 53.83)	39.29 (20.64 -57.95)	21.49 (4.38 - 38.60)	0.345

*Data are reported as mean (95% confidence interval).

§Data are reported as median (interquartile range Q 1-Q3).

^Hb,^ haemoglobin; ^GB,^ white blood cells; ^NEUT,^ neutrophils; ^LINF,^ lymphocytes; ^PLT,^ platelets; ^PCR,^ c-reactive protein; ^LDH,^ lactic dehydrogenase; ^IL-6,^ interleukin-6. Bold values means statistically significant.

**Table 4:** Comparison of the main comorbidities found in the four groups.

Variable	Group 1	Group 2	Group 3	Group 4	p
	↓NK (n=48)	↓CD4 (n=91)	CT (n=69)	↓PL C (n=40)	
Hypertension _(%)_	72.7	73.3	65.6	70.6	0.587
Obesity _(%)_	31.8	37.6	46.9	45.9	0.179
Diabetes Mellitus 2 _(%)_	29.5	32.6	35.9	41.2	0.625
MCVC _(%)_	25.0	37.2	34.4	23.5	0.132
CRI _(%)_	25.0	20.9	23.4	8.8	**0.026**

↓NK, reduction of natural killer lymphocytes; ↓CD4, reduction of helper T lymphocytes; ↓TC, reduction of cytotoxic T lymphocytes; ↓PLC, reduction of plasma cells. Bold values means statistically significant.

## Discussions

This study provides a detailed analysis of the immunological landscape in patients hospitalised with COVID-19, offering valuable insights into the interaction between innate and adaptive immunity during viral infections. Using multiparametric flow cytometry (MPFC), we identified specific immune phenotypes particularly decreasing inCD4^+^ and NK cell reductions which were independently associated with adverse clinical outcomes, including ICU admission and mortality. Importantly, these results not only support previous findings but also propose a reproducible framework for risk stratification based on immune signatures. The prognostic importance of reductions in CD4+ and NK cells is particularly significant. CD4+ T cells are crucial in coordinating immune responses by supporting B cell activity, regulating cytotoxic T lymphocytes, and maintaining cytokine balance. Their depletion, seen in 61.1% of our cohort, may suggest a state of immune weakness where patients are less capable of mounting a coordinated and effective defence against viral replication and secondary complications. Similarly, NK cells, vital components of innate immunity, enable early antiviral responses through cytolytic actions and cytokine production. A decline in NK cell counts, recorded in 32.2% of our patients, was associated with a threefold increase in the risk of ICU admission or death (OR 3.19, p<0.03). The combined deficiency of both CD4+ and NK cells, identified in 10% of patients, carried the highest risk, with a 9.5-fold rise in adverse outcomes independent of age and sex. These results reinforce previous reports that identified lymphopenia, particularly involving CD4+ and CD8+ T cells, as a hallmark of severe COVID-19 (5, 10, 23, 24). Our study also aligns with the work of Henry et al., Xu et al., and Carsetti et al., who described significant alterations in both innate and adaptive immunity in patients with worse prognosis (1, 6, 15). Furthermore, it builds upon our earlier findings, where we outlined distinct immune clusters among hospitalised patients and demonstrated a connection between multi-lineage lymphocyte depletion and mortality (19, 20). The value of this investigation lies in its ability to classify patients into biologically meaningful groups based on immune function. Instead of relying solely on clinical or radiological criteria, which may lag behind immunological deterioration, we propose a model that predicts risk through objective immunophenotyping. This approach opens a promising path towards personalised monitoring and decision-making. For example, patients with early decreases in CD4+ and NK cells may benefit from increased surveillance, pre-emptive supportive care, or targeted immunomodulation. Interestingly, reductions in CD8+ cytotoxic T cells and plasma cells, although observed in a significant proportion of patients (46% and 26.6%, respectively), did not reach statistical significance in predicting mortality or ICU transfer. While this may be due to the sample size or timing of immune sampling, it may also indicate that the orchestration of the immune response rather than the absolute number of specific effectors is the key factor in clinical progression. Alternatively, CD8+ dysfunction might be more relevant in the later stages of the disease or in post-viral syndromes such as long COVID, where persistent antigen exposure and immune exhaustion could play larger roles. From a pathophysiological perspective, our data support the idea of COVID-19 as an immunological disease with both hyperinflammatory and immunosuppressive aspects. Early in the pandemic, much focus was given to the “cytokine storm” hypothesis, highlighting the dysregulation of IL-6, IL-1β, and TNF-α. Although these remain important, our findings indicate that immune cell depletion, especially of key regulatory and cytotoxic subsets, may serve as a more insightful biomarker of deterioration. This view promotes a more nuanced understanding of the immune response: one that recognises both excessive activation and functional breakdown. Beyond the immediate context of COVID-19, this work adds to a growing body of literature advocating for immune profiling in managing infectious diseases. The MPFC methodology used here is rapid, standardised, and clinically applicable requiring only 2 ml of whole blood and providing results in less than an hour (18). This makes it an ideal candidate for integration into routine hospital workflows, especially in internal medicine wards caring for the elderly, with comorbidities, or immunosuppressed patients. The reproducibility of these immune signatures across different viral infections, including influenza and RSV, as well as post-vaccination adverse events, warrants further investigation (9). Furthermore, our findings could help for characterising immune responses in long COVID and other post-acute sequelae. Persistent immune dysregulation manifesting as T cell exhaustion, low NK cell activity, or poor antibody maturation has been proposed as a mechanism for ongoing fatigue, cognitive dysfunction, and inflammatory symptoms in convalescent patients (14, 25). The identification of early immune signatures during the acute phase may help predict which patients are at risk of developing chronic sequelae and guide early interventions.

This study has limitations. First of all it was a single-centre study in Palermo from March to April 2022. This short time and the observational design preclude causal inference restricting generalisability, especially in populations with varying vaccination coverage or different viral variants. However, although the study was conducted at a single centre, this design ensured methodological uniformity and consistent clinical management, which strengthens the internal validity of our findings and supports their potential reproducibility across different healthcare settings Secondly, All patients were hospitalised for moderate to severe COVID-19 (WHO clinical progression scale ≥ 4); vaccination data were not systematically available at the time of enrolment (early 2022) and have therefore been acknowledged as a study limitation; history of prior SARS-CoV-2 infection was not available for all subjects, and this has also been noted as a potential source of heterogeneity. The study design involved a single blood sampling at admission, and that no longitudinal follow-up was conducted to assess immune recovery or progression. Immune phenotyping was conducted at only one time point upon admission, with no longitudinal follow-up to assess immune recovery or ongoing dysfunction. Furthermore, potential confounders such as pre-existing immunosuppression, prior vaccination status, and viral variants were not stratified in the analysis. Morever, in this study beyond age and sex Key comorbidities (hypertension, diabetes, obesity, chronic kidney disease), were not fully adjusted for due to limited statistical power. Future research should involve multicentre data collection, repeated sampling over time, and genomic or cytokine profiling to deepen our understanding of immune responses and enhance predictive models. Nevertheless, the robustness of the observed associations, especially the significant independent predictive value of CD4+/NK+ depletion, supports the clinical value of this immunophenotypic framework. By combining easily measurable immune variables with clinical data, we can develop scalable tools to stratify risk, optimise resource allocation, and personalise treatment strategies not only for COVID-19 but also for a wide range of infectious and inflammatory syndromes. The identification of these cell subpopulations provide crucial insights into the immune determinants of vaccine response, potentially guiding the future development of tailored vaccination strategies or identifying individuals at particularly high risk in vulnerable population or in elderly patients admitted to internal medicine ward. Given this background personalised medicine could customize medical interventions based on a person’s unique genetic makeup, lifestyle, and medical history. The real-time feasibility of multiparametric immunophenotyping could integrated into precision internal medicine workflows. By embedding immunophenotypic data into digital platforms for patient stratification, therapeutic monitoring, and early detection of subclinical disease, clinicians can move beyond static diagnostic paradigms toward adaptive, individualized care models. Such integration not only enhances diagnostic accuracy and therapeutic responsiveness but also establishes immunophenotyping as a pivotal tool in the operationalization of precision medicine across diverse internal medicine contexts. In conclusion, our findings confirm that hospitalised patients with COVID-19 display diverse immune profiles that are strongly linked to clinical outcomes. Specifically, decreases in CD4+ T helper and NK cells form a high-risk phenotype with independent prognostic significance. These results highlight the importance of incorporating immunological monitoring into clinical pathways and support a broader shift towards precision immunology in patients affected by viral or immuno-inflammatory diseases. By recognising immune phenotypes as dynamic markers of vulnerability, we may improve patient care not by reacting to deterioration, but by predicting and preventing it.

## Data Availability

The original contributions presented in the study are included in the article/supplementary material. Further inquiries can be directed to the corresponding author.

## References

[1] Henry BM de Oliveira MHS Benoit S Plebani M Lippi G . Hematologic, biochemical and immune biomarker abnormalities associated with severe illness and mortality in COVID-19: a meta-analysis. Clin Chem Lab Med. (2020) 58:1021–8. doi: 10.1515/cclm-2020-0369, PMID: 32286245

[2] Chowdhury MA Hossain N Kashem MA Shahid MA Alam A . Immune response in COVID-19: A review. J Infect Public Health. (2020) 13:1619–29. doi: 10.1016/j.jiph.2020.07.001, PMID: 32718895 PMC7359800

[3] Thevarajan I Nguyen THO Koutsakos M Druce J Caly L van de Sandt CE . Breadth of concomitant immune responses prior to patient recovery: a case report of non-severe COVID-19. Nat Med. (2020) 26:453–5. doi: 10.1038/s41591-020-0819-2, PMID: 32284614 PMC7095036

[4] Zheng HY Zhang M Yang CX Zhang N Wang XC Yang XP . Elevated exhaustion levels and reduced functional diversity of T cells in peripheral blood may predict severe progression in COVID-19 patients. Cell Mol Immunol. (2020) 17:541–3. doi: 10.1038/s41423-020-0401-3, PMID: 32203186 PMC7091621

[5] Diao B Wang C Tan Y Chen X Liu Y Ning L . Reduction and functional exhaustion of T cells in patients with coronavirus disease 2019 (COVID-19). Front Immunol. (2020) 11:827. doi: 10.3389/fimmu.2020.00827, PMID: 32425950 PMC7205903

[6] Xu B Fan CY Wang AL Zou YL Yu YH He C . Suppressed T cell-mediated immunity in patients with COVID-19: A clinical retrospective study in Wuhan, China. J Infect. (2020) 81:e51–60. doi: 10.1016/j.jinf.2020.04.012, PMID: 32315725 PMC7166040

[7] Lucas C Wong P Klein J Castro TBR Silva J Sundaram M . Longitudinal analyses reveal immunological misfiring in severe COVID-19. Nature. (2020) 584:463–9. doi: 10.1038/s41586-020-2588-y, PMID: 32717743 PMC7477538

[8] Sun DW Zhang D Tian RH Li Y Wang YS Cao J . The underlying changes and predicting role of peripheral blood inflammatory cells in severe COVID-19 patients: A sentinel? Clin Chim Acta. (2020) 508:122–9. doi: 10.1016/j.cca.2020.05.027, PMID: 32417210 PMC7224669

[9] Jiang M Guo Y Luo Q Huang Z Zhao R Liu S . T-cell subset counts in peripheral blood can be used as discriminatory biomarkers for diagnosis and severity prediction of COVID-19. J Infect Dis. (2020) 222:198–202. doi: 10.1093/infdis/jiaa252, PMID: 32379887 PMC7239156

[10] Qin R He L Yang Z Jia N Chen R Xie J . Identification of parameters representative of immune dysfunction in patients with severe and fatal COVID-19 infection: a systematic review and meta-analysis. Clin Rev Allergy Immunol. (2023) 64:33–65. doi: 10.1007/s12016-021-08908-8, PMID: 35040086 PMC8763427

[11] Liu J Li S Liu J Liang B Wang X Wang H . Longitudinal characteristics of lymphocyte responses and cytokine profiles in the peripheral blood of SARS-CoV-2 infected patients. EBioMedicine. (2020) 55:102763. doi: 10.1016/j.ebiom.2020.102763, PMID: 32361250 PMC7165294

[12] Adhikari A Maddumage J Eriksson EM Annesley SJ Lawson VA Bryant VL . Beyond acute infection: mechanisms underlying post-acute sequelae of COVID-19 (PASC). Med J Aust. (2024) 221 Suppl 9:S40–8. doi: 10.5694/mja2.52456, PMID: 39489518

[13] Sette A Crotty S . Adaptive immunity to SARS-Cov-2 and COVID-19. Cell. (2021) 184:861–80. doi: 10.1016/j.cell.2021.01.007, PMID: 33497610 PMC7803150

[14] Rezaei M Mahmoudi S Mortaz E Marjani M Tabarsi S Ziai SA . Immune cell profiling and antibody responses in patients with COVID-19. BMC Infect Dis. (2021) 21:646. doi: 10.1186/s12879-021-06278-2, PMID: 34225645 PMC8256640

[15] Carsetti R Zaffina S Piano Mortari E Terreri S Corrente F Capponi C . Different innate and adaptive immune responses to SARS-CoV-2 infection of asymptomatic, mild, and severe cases. Front Immunol. (2020) 11:610300. doi: 10.3389/fimmu.2020.610300, PMID: 33391280 PMC7772470

[16] Neirinck J Buysse M De Vriendt C Hofmans M Bonroy C . The role of immunophenotyping in common variable immunodeficiency: a narrative review. Crit Rev Clin Lab Sci. (2024) 62:65–84. doi: 10.1080/10408363.2024.2404842, PMID: 39364936

[17] Sun Y Dong Y Wang L Cui G Yu Z Ren Z . Immune response induced by novel coronavirus infection. Front Cell Infect Microbiol. (2022) 12:988604. doi: 10.3389/fcimb.2022.988604, PMID: 36389144 PMC9641212

[18] Finak G Langweiler M Jaimes M Malek M Taghiyar J Korin Y . Standardizing flow cytometry immunophenotyping analysis from the Human ImmunoPhenotyping Consortium. Sci Rep. (2016) 6:20686. doi: 10.1038/srep20686, PMID: 26861911 PMC4748244

[19] Corrao S Gervasi F Di Bernardo F Natoli G Raspanti M Catalano N . Immunological characteristics of non-intensive care hospitalized COVID-19 patients: a preliminary report. J Clin Med. (2021) 10:849. doi: 10.3390/jcm10040849, PMID: 33669527 PMC7921979

[20] Corrao S Gervasi F Di Bernardo F Argano C . Immune response failure in paucisymptomatic long-standing SARS-CoV-2 spreaders. Clin Pract. (2021) 11:151–61. doi: 10.3390/clinpract11010021, PMID: 33804326 PMC7930978

[21] Bocsi J Melzer S Dähnert I Tárnok A . OMIP-023: 10-color, 13 antibody panel for in-depth phenotyping of human peripheral blood leukocytes. Cytometry A. (2014) 85:781–4. doi: 10.1002/cyto.a.22505, PMID: 25132115

[22] De Pace V Bruzzone B Orsi A Ricucci V Domnich A Guarona G . Comparative analysis of five multiplex RT-PCR assays in the screening of SARS-CoV-2 variants. Microorganisms. (2022) 10:306. doi: 10.3390/microorganisms10020306, PMID: 35208761 PMC8876857

[23] Vivier E Raulet DH Moretta A Caligiuri MA Zitvogel L Lanier LL . Innate or adaptive immunity? The example of natural killer cells. Science. (2011) 331:44–9. doi: 10.1126/science.1198687, PMID: 21212348 PMC3089969

[24] Zheng M Gao Y Wang G Song G Liu S Sun D . Functional exhaustion of antiviral lymphocytes in COVID-19 patients. Cell Mol Immunol. (2020) 17:533–5. doi: 10.1038/s41423-020-0402-2, PMID: 32203188 PMC7091858

[25] Fergie J Srivastava A . Immunity to SARS-CoV-2: lessons learned. Front Immunol. (2021) 12:654165. doi: 10.3389/fimmu.2021.654165, PMID: 33815415 PMC8018176

